# Correlation between use of different type protective facemasks and the oral ecosystem

**DOI:** 10.1186/s12889-023-16936-6

**Published:** 2023-10-12

**Authors:** Simonetta D’Ercole, Paolo Parisi, Sara D’Arcangelo, Felice Lorusso, Luigina Cellini, Tatiane Cristina Dotta, Maristella Di Carmine, Morena Petrini, Antonio Scarano, Domenico Tripodi

**Affiliations:** 1 Department of Medical, Oral and Biotechnological Sciences, University “G. D’Annunzio” of Chieti- Pescara, Via dei Vestini, 31, Chieti, 66100 Italy; 2 Department of Dental Materials and Prosthodontics, School of Dentistry of Ribeirão Preto, University of São Paulo, São Paulo, 14040-904 Brazil; 3 Department of Pharmacy, University “G. D’Annunzio” of Chieti-Pescara, Via dei Vestini, 31, Chieti, 66100 Italy; 4 Department of Innovative Technologies in Medicine and Dentistry, University “Gd’Annunzio” of Chieti-Pescara, Via dei Vestini, 31, Chieti, 66100 Italy

**Keywords:** COVID-19, Face mask, FFP2 mask, Oral cavity, Oral ecosystem, PPE

## Abstract

**Background:**

Spread worldwide through droplets, the Virus Sars-Cov-19 has caused a global health emergency alarm. In order to limit its spread, the use of masks has become part of the daily life of the entire population, however, little is known about its constant use and the changes generated in the oral cavity. This work aims to investigate correlations between the continuous use of masks covering the nose and mouth for 3 h and changes in the ecological factors of the oral cavity.

**Methods:**

34 volunteers were divided into 2 groups: wear only the filtering facepiece code 2 (FFP2) mask (Group A) and wear the FFP2 mask covered by a surgical mask (Group B). Measurement of Volatile Organic Compounds (VOCs), saliva rehydration and consistency test, collection of basal saliva and saliva stimulated with paraffin gum and mucosal swab were collected and analyzed at two times: before using the mask(s) (T_0_) and 3 h after continuous use of the mask(s) (T_1_).

**Results:**

The results indicated a significant difference between the groups, in which the basal saliva volume and pH and the peaks of VOCs increased for group B between T_0_ and T_1_. The rehydration time decreased and the volume and pH of the stimulated saliva increased, but with no significant difference between the groups. Furthermore, group B showed a significant decrease in *Candida albicans* Colony Forming Units (CFUs) and Total Bacterial Count (TBC) between T_0_ and T_1_.

**Conclusion:**

It is concluded that the prolonged use of the FFP2 mask covered by a surgical mask can generate oral alterations in the user.

## Background

Virus Sars-Cov-19 spread rapidly by droplets in several countries in 2020, activating the global health emergency alarm [1]. To limit its dissemination, governments imposed the use of personal protective equipments (PPE), such as face shields, masks, and, in many countries, filtering facepiece code 2 (FFP2) covered by a surgical mask during medical treatment, becoming part of our daily lives and playing an important and preponderant role in health care, mainly involving doctors, dentists and all health professionals [2].

Several studies have been carried out to assess the effectiveness of masks and their filtering capacity, even in years before the Era of COVID-19 [2–5]. An essential premise for these studies involves the analysis of the different types of PPE, its characteristics and differences, and the filtering effectiveness.

The efficacy of the mask’s barrier function can be measured by different test. The Fit-test represents the “control of adaptability to the face” and consists of a series of tests per sample, intended to verify the suitability of the PPE for health professionals, being useful in cases where compliance is desired of a batch of FFP2 masks purchased by the employer for his hospital staff. On the other hand, the Fit-check is the “individual fit control” and consists of simple maneuvers to use and review the fit of the individual mask [6, 7].

The tightness of the seal created by these masks is a factor of primordial importance to standardize the experimentation and, above all, to support the thesis that the FFP2 masks favor the increase of the concentration of carbon dioxide (CO_2_) in the oral cavity, causing a variation in the oral ecosystem, mainly when covered with a surgical mask [8, 9].

There is a scarcity of articles in the literature that verifies the alteration of the oral ecosystem after prolonged use of PPE. However, there are numerous articles related to the discomfort arising from them, physical and mental problems, and systemic changes in the concentration of oxygen (O_2_) [8–11]. Face masks cause a flow resistance that raises the temperature of the face at the site, which can trigger a panic disorder caused by high levels of CO_2_ under the mask, with hot flashes and sweating [10, 12]. In addition to an increase in heart rate, shortness of breath, dizziness, and headache [11].

The oral ecosystem comprises oral microorganisms set and is an “open system”, as microbes and protective factors are continuously inserted and removed even with simple breathing [13]. The oral microbiome has a commensal or symbiotic relationship with the host, and maintaining a eubiotic balance ensures a healthy oral cavity [14, 15]. The variation of CO_2_ inevitably leads to a situation of dysbiosis, as it favors anaerobic bacterial species to the detriment of aerobic ones [13].

An increase in CO_2_ concentrations lowers the pH making it more acidic, as the gas, combined with water (H_2_O), produces carbonic acid (H_2_CO_3_) by chemical reaction [13, 16]. According to Stephan’s theory, the saliva represents an excellent defense system because, thanks to the substances dissolved in it and the buffer system, it neutralizes pH variations [16].

Furthermore, saliva reflects oral health and provides useful information about the microbiota, so an analysis of the main ecological factors of the oral cavity (saliva volume, pH, Volatile Organic Compounds-VOCs) allows a complete picture of the health/disease condition of the oral cavity [17].

VOCs are carbon-based chemical compounds capable of evaporating easily at room temperature and pressure [18]. This category includes alcohols, aliphatic hydrocarbons, aromatic hydrocarbons, aldehydes, ketones, esters, halogenated hydrocarbons, and CO_2_. For example, an assessment of these molecules in the expired breath, permit objectively to evaluate halitosis. Bad breath is mainly the result of bacterial degradation of salivary mucoproteins, food debrides, and plaque deposits that lead to the production of increased levels of hydrogen sulfide, methyl mercaptan, dimethyl sulfide, and carbon dioxide [19]. Both gram-positive and negative bacteria can contribute to the production of these molecules, and halitosis can affect patients of all ages, with or without oral pathologies [20, 21].

Salivary tests are useful to assess oral ecosystem conditions, such as rehydration times, saliva composition, amount of basal and stimulated saliva, and its pH, directly in the dental office [19]. Changes in the balance of the oral microbiota cause different infectious diseases, with the presence of Gram-positive and negative aerobic microorganisms that lead to caries lesions and periodontal disease [22].

Based on these premises, this work aims to investigate correlations between the continuous use of face masks covering the nose and mouth for 3 h and changes in the ecological factors of the oral cavity. The null hypothesis was that there were no differences in terms of changes in oral ecological factors if the two different ways of wearing masks (FFP2 mask only and FFP2 mask covered by surgical mask) covered nose and mouth for the same time of 3 h. The alternative hypothesis was that the two different ways of wearing masks affected in positive/negative way the state of oral cavity.

## Methods

### Design, sample, data collection

34 students specialized in Pediatric Dentistry and interns in Dentistry from the “G. d’Annunzio” University of Chieti-Pescara were recruited (17 men and 17 women).

They were randomly divided into 2 groups:

- Group A: 17 volunteers wearing only the FFP2 mask.
- Group B: 17 volunteers wearing the FFP2 mask covered by a surgical mask.

Inclusion criteria: individuals between 24 and 38 years old, healthy systemically and dentally, and without any disease.

Exclusion criteria: the presence of diabetes, autoimmune diseases, xeroderma, pregnancy, current infections, serious chronic diseases, hormonal therapies, severe kidney or liver failure, hyper or hypothyroidism, drug or alcohol consumption, general systemic diseases. In addition, individuals with extensive and multiple caries lesions, any form of lichen, leukoplakia or erythroplakia, and periodontal patients.

The study was conducted according to the Declaration of Helsinki to protect human research subjects. The selected subjects voluntarily participated in the study after receiving oral and written information on the purpose of the research. The written informed consent was signed by the participants (Privacy Law DL 196/2003).

Before starting the study, the volunteers were properly trained on the use of masks and the importance of the correct fit (Fit-check). To allow adequate adhesion of the FFP2 mask to the face, the procedures proposed by the Italian National Institute for Occupational Accident Insurance (INAIL) were illustrated with the dressing phase followed by the Fit-check (adaptation control). This ensured the device was sealed as tightly as possible according to medical and manufacturer standards.

Study participants received the following indications:

- Do not wear any type of mask before the start of the assessment. If necessary, stick to the use of a disposable surgical mask;
- Refrain from taking food and smoking in the hours before the visit/collection;
- Refrain from food and smoking during experimentation;
- Do not touch or limit the movement of the mask(s) to a minimum to maintain a seal;
- During the experiment, it was strictly forbidden to remove the mask(s), except for drinking water.

The masks used were FFP2 NR Value & Safe, model LK-Z1510, imported by Gima S.p.a. (Gessate, MI), LOTE number 102B105 and European certification CE 2163, complying with EU standards: 2016/425 and EN 149: 2001 + A1: 2009.

Two observation times were distinguished:

- T_0_: before putting on the mask(s).
- T_1_: 3 h after continuous use of the mask(s).

In T_0_, a medical record was compiled containing a complete medical and dental history, with an attached description of the volunteer’s diet. Then, an intraoral examination of the mucosa and hard tissues with related bad habits, parafunctions, joint problems, occlusion, and DMFT (decayed, missing and filled dental) index was performed.

After this first phase, at T_0_ and T_1_ were performed:

1. measurement of VOCs;
2. saliva rehydration;
3. basal saliva collection;
4. collection of saliva stimulated with paraffin gum;
5. mucosal swab.

The quantitative determination (ml/5 min) of basal and stimulated saliva was performed and a chairside kit for saliva pH evaluation was adopted (Chairside Saliva-Check Buffer® from GC Europe in Leuven, Belgium). The collection and use of saliva were approved by the Ethics Committee of University “G. d’Annunzio”, Chieti-Pescara, Italy (approval code SALI, N. 19 of the 10 September 2020).

Stimulated saliva and mucosal swab were sent to the microbiology laboratory for microbiological evaluation.

### Volatile organic compounds (VOCs) measurement

**Fig. 1:**
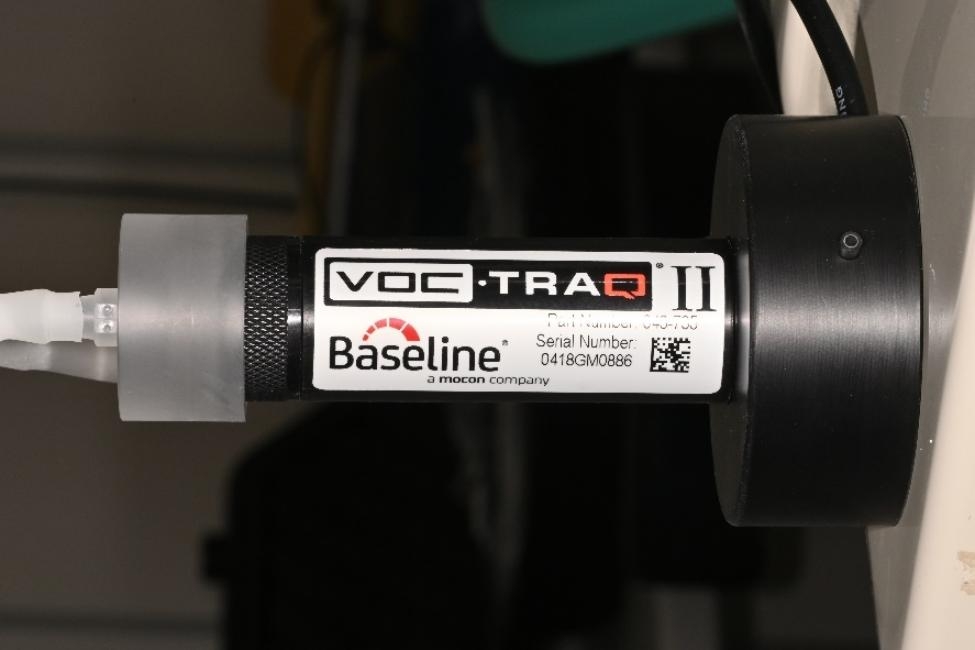
Integrative VOC sensor and pump device

Real-time measurements of VOCs were performed at T_0_ and T_1_ at the same location in the operating room under standardized temperature conditions (21 °C ± 1). A dedicated electronic sensor was used for the survey (TRAQ-II, Mocon, Lyons, CO, USA) [23] (Fig. 1). The device is specific for air detecting of organic and inorganic compounds including alcohols, aliphatic hydrocarbons, volatile sulfur compounds, aldehydes, aromatic hydrocarbons, amines, ketones, organic acids and CO_2_ [23]. For further statistical methods, the maximum VOC mean peaks were considered. The device calibration was performed in accordance to the manufacturer’s indications and a minimum of 10 min for washing-out period was observed between each measurement [23]. This device was connected to a sterile disposable tube connected to a pump to obtain the saturation of the sensor device [23]. The measurement procedure was performed by imposing slow breathing maneuvers and repeated for three times. The highest peak level was considered for statistical analysis.

### Salivary rehydration

**Fig. 2:**
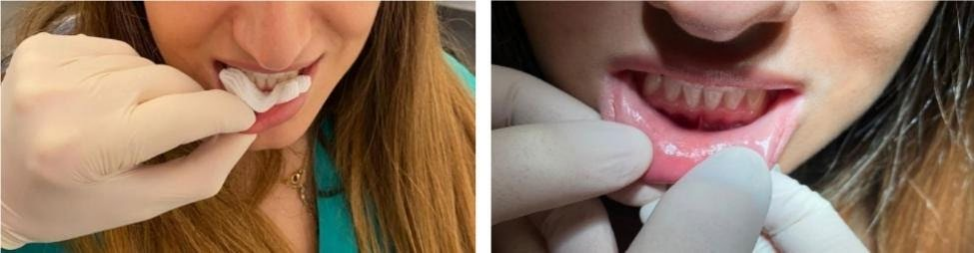
Hands-on demonstration of the rehydration test

For the salivary rehydration the volunteer’s lower lip was exposed, and the fornix was dried with a sterile gauze, holding the lip with the fingers (Fig. 2). Fornix rehydration time and lip droplet formation were timed, recording the results in special tables.

### Basal saliva collection: expectoration technique/spitting method

The volunteer was instructed to keep the oral floor leaning forward and was asked to collect his saliva in a graduated glass whenever she felt like swallowing. The test lasted 5 min, after which data on the amount in ml of saliva was collected, and the pH was evaluated with a detection kit.

### Saliva collection stimulated with paraffin gum

The paraffin gum (CRT® Bacteria - Ivoclar Vivadent AG - Schaan, Liechtenstein) was removed with sterile forceps and taken to the volunteer’s mouth, who chewed it for 5 min. The first saliva of the first 20 s was expelled in the sink, while the rest was collected in the graduated test tube.

Also in this case, the volume of saliva in ml was evaluated, and the pH was calculated with a detection kit.

### Mucosal smear

**Fig. 3:**
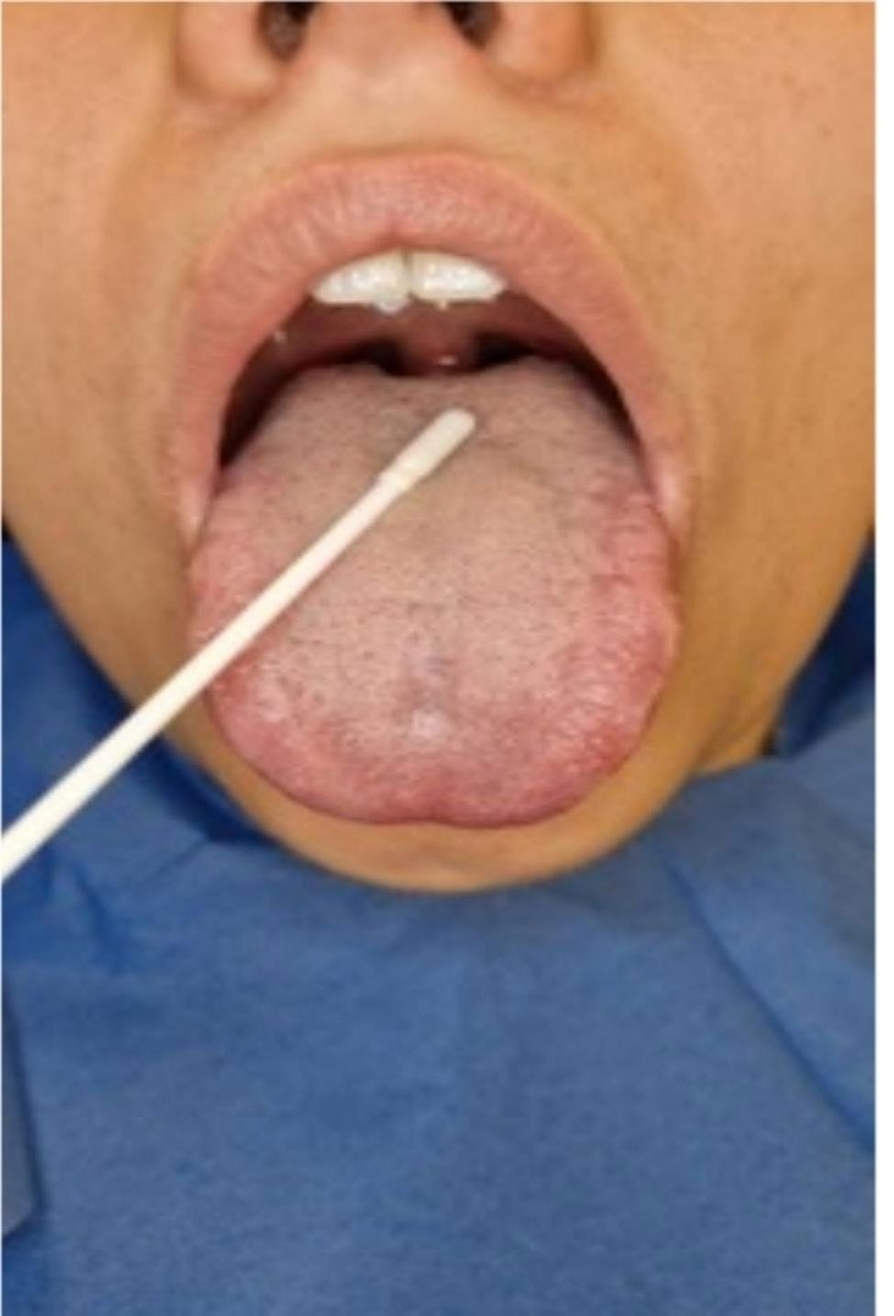
Sampling of the mucous membranes with a cotton swab

The swab was spread on the mucous membranes of the oral cavity, particularly in the retrolabial commissures and on the deep dorsum of the tongue (Fig. 3). The swab was then stored in the tube containing 1 ml of transport medium, closed, and sent to the microbiology laboratory.

### pH test

The pH test was performed for basal and stimulated saliva. A part of the special paper was removed with university tweezers and immersed in the salivary samples, in which the color change occurred according to the pH. Subsequently, the color obtained was compared with the chromatic scale of the kit, where a pH value is associated with each color. Finally, the data were collected in tables.

### Determination of total bacterial Count in stimulated saliva

Saliva stimulated with paraffin gum, collected in a test tube at T_0_ and T_1_, was sent to the microbiology laboratory to determine the aerobic Total Bacterial Count (TBC).

TBC was enumerated applying the plate counting method.

The initial sample, properly diluted in serial dilutions with DPBS (Dulbecco’s Phosphate Buffered Saline) diluent, was placed on agar plates containing TSA (Tryptic Soy Agar), using the surface plate method. Replicate plates were labeled, cultured and incubated for 48 h at 37 °C. TBC was obtained by counting visible distinct colonies using the colony counter and calculated as Colony Forming Unit (CFU) of the sample and correlated based on the dilution factor.

### Determination of fungal cells on swab test

All mucosal swab specimens underwent fungal enrichment by inoculation into RPMI 1640 (L-glutamine and sodium bicarbonate medium) supplemented with 2% glucose, an enrichment to promote *Candida* growth, were incubated at 37 °C for 24 h and then visually examined for turbidity. Subsequently, the samples were immediately cultured on SDA culture medium (Sabouraud Dextrose Agar), useful for the cultivation and maintenance of pathogenic and non-pathogenic species of fungi and yeasts.

After incubation for 24 h at 37 °C, isolates, grown as yeast on SDA, were identified by the morphological appearance of the colonies for typical *Candida* spp. Then, single colonies were struck on SDA, incubated for 24 h at 37 °C, and pure cultures were performed from those. After subculturing, pure cultures were biochemically characterized using API® 20CAUX yeast galleries (bioMérieux Italia), prepared according to the manufacturer’s instructions.

### Stastistical analysis

The evaluation of the homogeneity of the groups was analyzed using the Levene test. The differences between the groups were statistically analyzed using the T-Test. The intragroup variation between T_1_ and T_0_ (T_1_-T_0_) was calculated for each parameters and then the averagy mean variation ± standard deviation was determined.

Statistically significant differences were considered to be a p-value < 0.05. The statistical software used to run these tests was SPSS statistical analysis software (IBM SPSS, Chicago, IL, USA).

## Results

During the study, two of the participants was excluded from group B, due to discomfort and difficulty in prolonged mask use.

In total, therefore, the results are based on 32 participating volunteers:

- Group A, 11 women and 6 men with a mean age of 29.36 ± 4.09 years;
- Group B, 6 women and 9 men with a mean age of 25.8 ± 3.30 years.

In total, among all participants, 4 smoked less than one pack of cigarettes per day, 1 in group A and 3 in group B. In addition, 4 volunteers were undergoing orthodontic treatment with aligners, 2 for each group, who were instructed not to use them during the experiment.

As far as nutrition is concerned, almost all of them followed the Mediterranean diet. Among them 2 had a diet that preferred vegetables over meat. Among the volunteers, 3 had an unbalanced diet correlated with snacks and junk food, while only 1 followed a hypocaloric diet combined with sports training.

Groups A and B had a mean DMFT of 2.41 ± 1.37 and 2.33 ± 1.04, respectively.All participants were instructed on oral hygiene care, and in fact, no significant amounts of tartar or plaque were found during the oral physical examination.

Among all participants, 15.6% had enamel caries in a single tooth, 16 participants had 2 restorations, 7 had 3 restorations, 2 had 4 and only 1 patient had a DMFT index of 6 due to edentulism of all first premolars by orthodontic reasons.

Volunteers with enamel caries were 3 for group A and 2 for group B, while the mean number of restorations was 2 ± 0.79 and 2.13 ± 0.91, respectively.

Third molar extractions were not considered in the DMFT index, respecting the WHO guidelines (Oral Health Surveys - Basic Methods World Health Organization, 2013).

The demographic characteristics of the 2 analyzed groups are presented in Table 1.

**Table 1:** Anamnestic data and DMFT index

	GROUP A		GROUP B	
GENDER	11 F	6 M	6 F	9 M
AGE	29.36 ± 4.09		25.8 ± 3.30	
SMOKING	5.9% (1/17)		20% (3/15)	
ALIGNERS	11.8% (2/17)		13.3% (2/15)	
DMFT INDEX	2.41 ± 1.37		2.33 ± 1.04	

Table 2 shows the VOC parameters of each group comparison at T_0_ and T_1_, while the average of the maximum peaks, the standard deviation, the minimum value of 95% CI (Confidence Interval) of the mean, and the maximum value of 95% CI have been reported.

At T_0_, groups A and B had mean peak maximum VOCs of 2.8 ± 1.0 and 3.1 ± 1.3 ppm, log_10_, respectively, with no significant difference (p > 0.05). At T_1_, group A had a mean VOC peak of 3.2 ± 1.5 ppm, log_10_, while group B reported 5.1 ± 2.2 ppm, log_10_ with a significant difference between the groups (p < 0.05).

The difference between the averages of the peaks of the T_0_/T_1_ VOCs indicates that the combination of the two masks has a greater influence on organic and inorganic compounds, unlike using the FFP2 mask alone.

**Table 2:** Peak VOCs (ppm, log_10_) and comparison between groups A and B at T_0_ (baseline) and T_1_ (after 3 h of use) [mean, standard deviation, lowest value 95% CI, highest value 95% CI, significance level]

VOCs Max peak measurements	T_0_		T_1_	
	**A**	**B**	**A**	**B**
Number of values	17	15	17	15
Mean	2.8	3.1	3.2	5.1
Std. Deviation	1.0	1.3	1.5	2.2
Lower 95% CI of mean	2.3	2.8	3	3.6
Upper 95% CI of mean	3.4	4.2	4.5	6.1
*P value*	p > 0.05		p < 0.05	

t-Student test

No statistically significant differences have been found for reydratation timing in the two groups at T_0_.

**Fig. 4:**
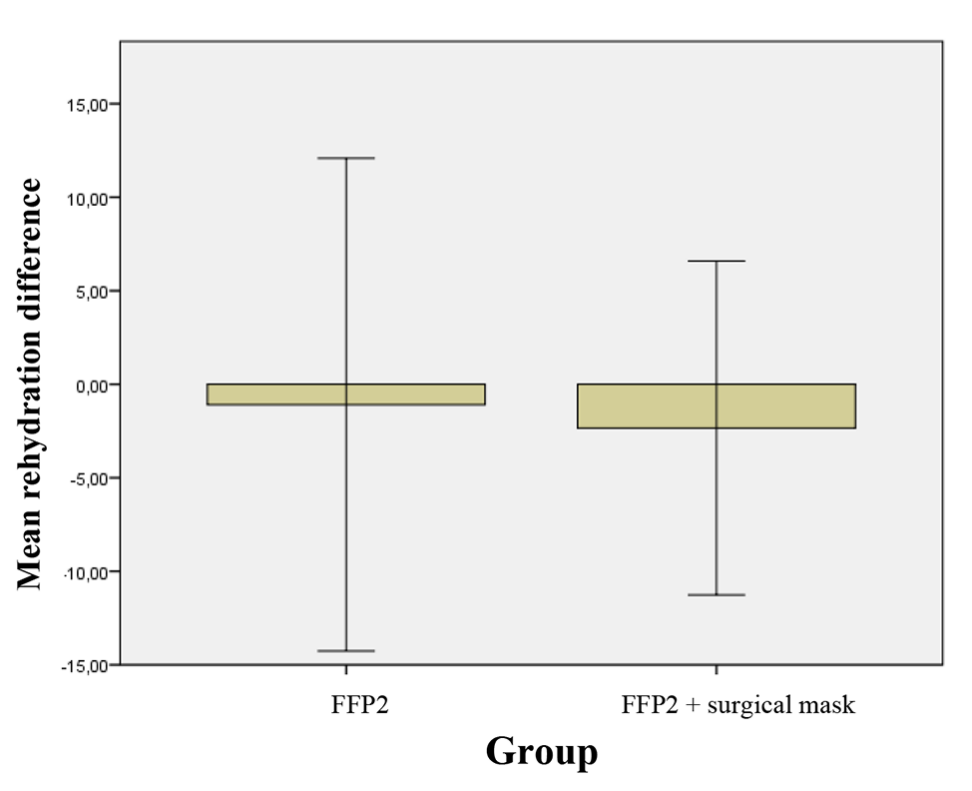
Mean of difference of T_1_-T_0_ and relative standard deviation of the rehydration in 2 groups

Rehydration times decreased in both groups A and B, although not statistically significant results were found (Table 3; Fig. 4).

**Table 3:** Data related to group A and B

	Group A		Group B	
	**T**_**0**_	**T**_**1**_	**T**_**0**_	**T**_**1**_
REHYDRATION TIME (s)	55.12 ± 20.28	50.18 ± 23.62	50.13 ± 13.39	40.93 ± 10.71
BASAL SALIVA VOLUME (ml/5min)	2.91 ± 1.28	2.95 ± 1.28	2.55 ± 1.07	3.59 ± 1.78
BASAL SALIVA pH	7.25 ± 0.41	7.35 ± 0.38	6.91 ± 0.6	7.13 ± 0.51
STIMULATE SALIVA VOLUME (ml/5min)	6.14 ± 3.4	6.87 ± 3.4	8.21 ± 3.43	8.64 ± 2.95
STIMULATED SALIVA pH	7.61 ± 0.29	7.75 ± 0.09	7.33 ± 0.5	7.57 ± 0.47

**Fig. 5:**
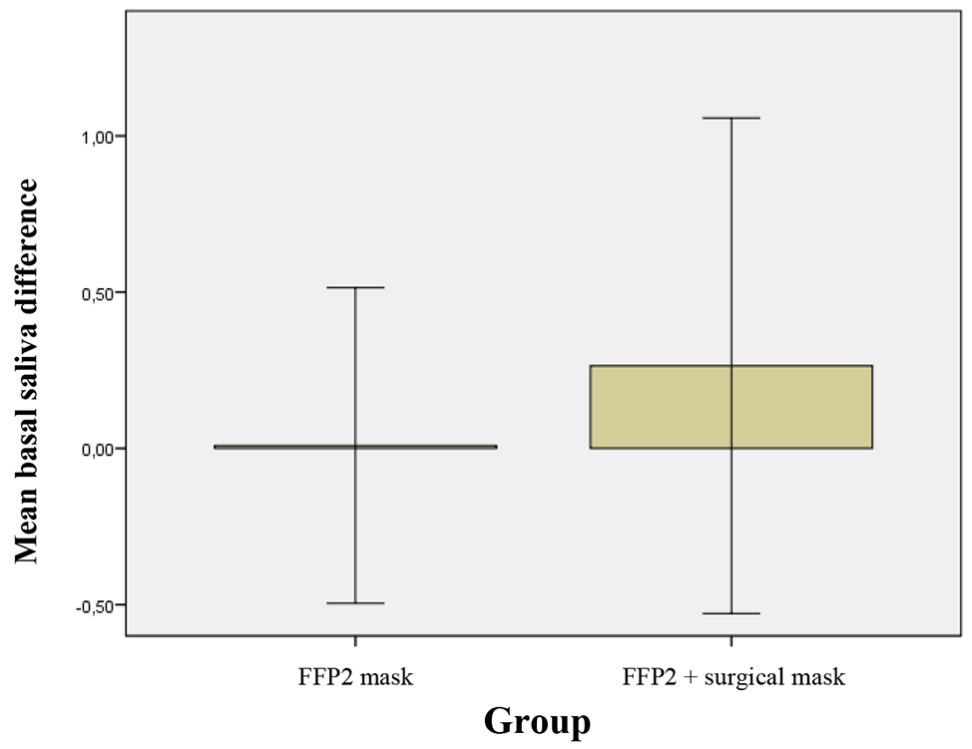
Mean of difference of T_1_-T_0_ and relative standard deviation of the basal saliva in 2 groups

The use of the FFP2 mask (group A) did not affect the production, determined in ml/5 min, of basal saliva. On the contrary, the simultaneous use of FFP2 and surgical masks (group B) significantly increases the amount of basal saliva. The difference between the T_0_/T_1_ avarage indicates that the combination of the two masks affects the basal saliva volume, unlike using the FFP2 mask alone (Table 3; Fig. 5). The different variation of basal saliva in group B respect group A was statistically significant, p = 0.034. At T0 no significant differences of basal saliva were present between group A and B.

**Fig. 6:**
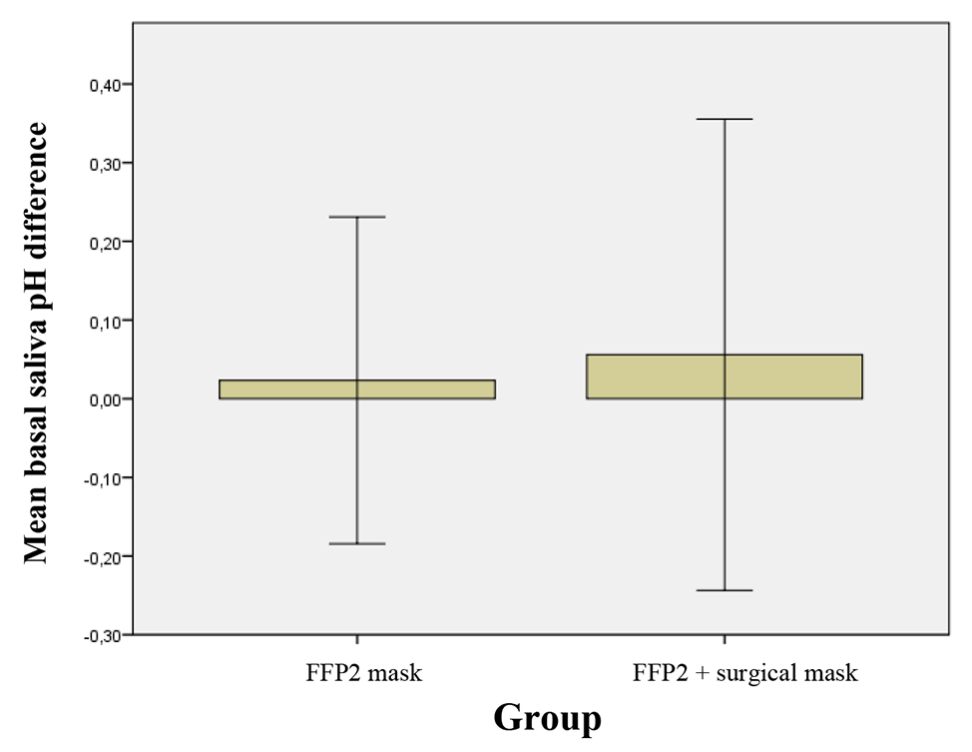
Mean of difference of T_1_-T_0_ and relative standard deviation of the basal saliva pH in 2 groups

The pH of the basal saliva remained almost constant in groups A and B (Table 3; Fig. 6). The difference between the T_0_/T_1_ means indicates that the combination of the two masks affects the pH of the basal saliva, unlike the use of the FFP2 mask alone, even if differently not statistically significant.

**Fig. 7:**
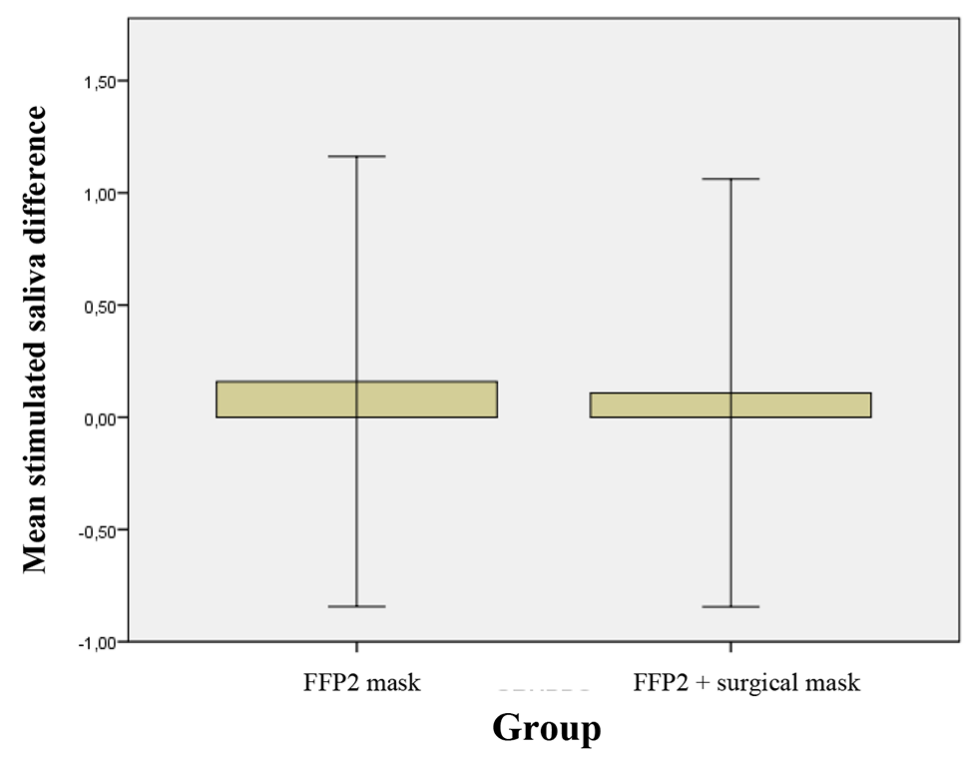
Mean of difference of T_1_-T_0_ and relative standard deviation of the stimulated saliva in 2 groups

Although the average volume of stimulated saliva was higher in group B than group A, no significant differences were found between the two groups at the T_0_. The volume of stimulated saliva increased in both groups A and B, although without statistical significance (Table 3; Fig. 7).

A significant difference in the baseline values of stimulated saliva between group A and B pH was already present at the T0, p = 0.011. In group A, the pH was 7.611 ± 0.875, and in group B 7.327 ± 0.498. At T1 the values of group A were 7.753 ± 0.875 and 7.573 ± 0.473. A greater increase was recorded in group B, but these variations were not statistically significant.

**Fig. 8:**
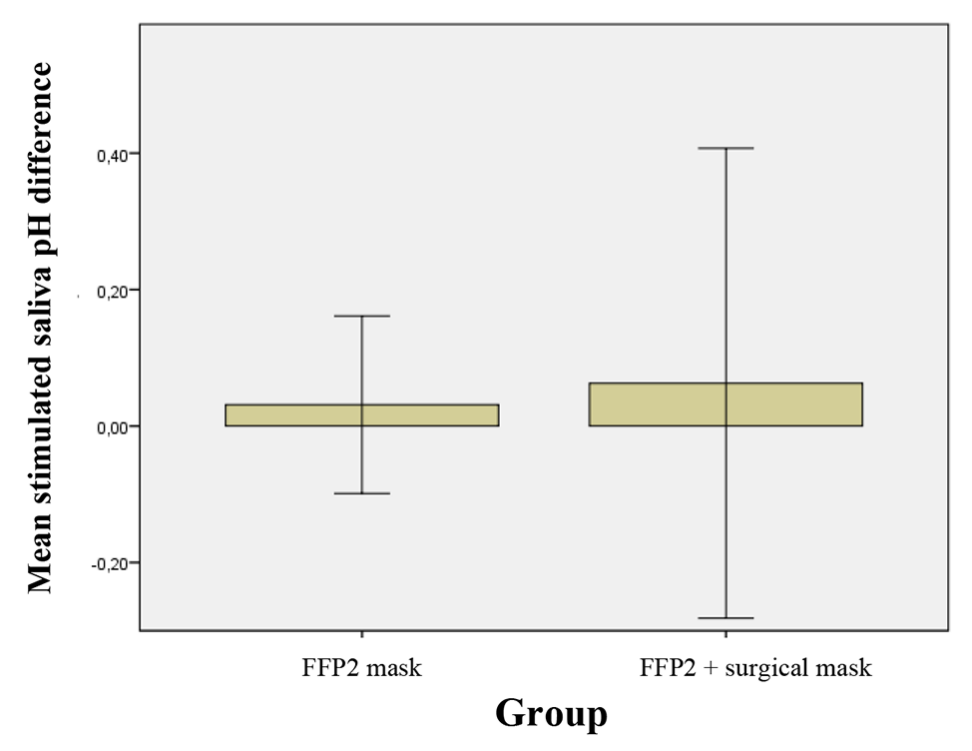
Mean of difference of T_1_-T_0_ and relative standard deviation of the stimulated saliva pH in 2 groups

The pH of the stimulated saliva remains almost constant in groups A and B (Fig. 8). The difference between the T_0_/T_1_ means indicates that the combination of the two masks affects the pH of the stimulated saliva, unlike the use of the FFP2 mask alone, even if not statistically significant.

At T_0_ a significant differences between TBC in group A and B were present (p < 0.001). These differences remained significant also at the T1 (p < 0.001). The variation between T1 and T0 in group A and B was statistically significant (p = 0.012).

**Fig. 9:**
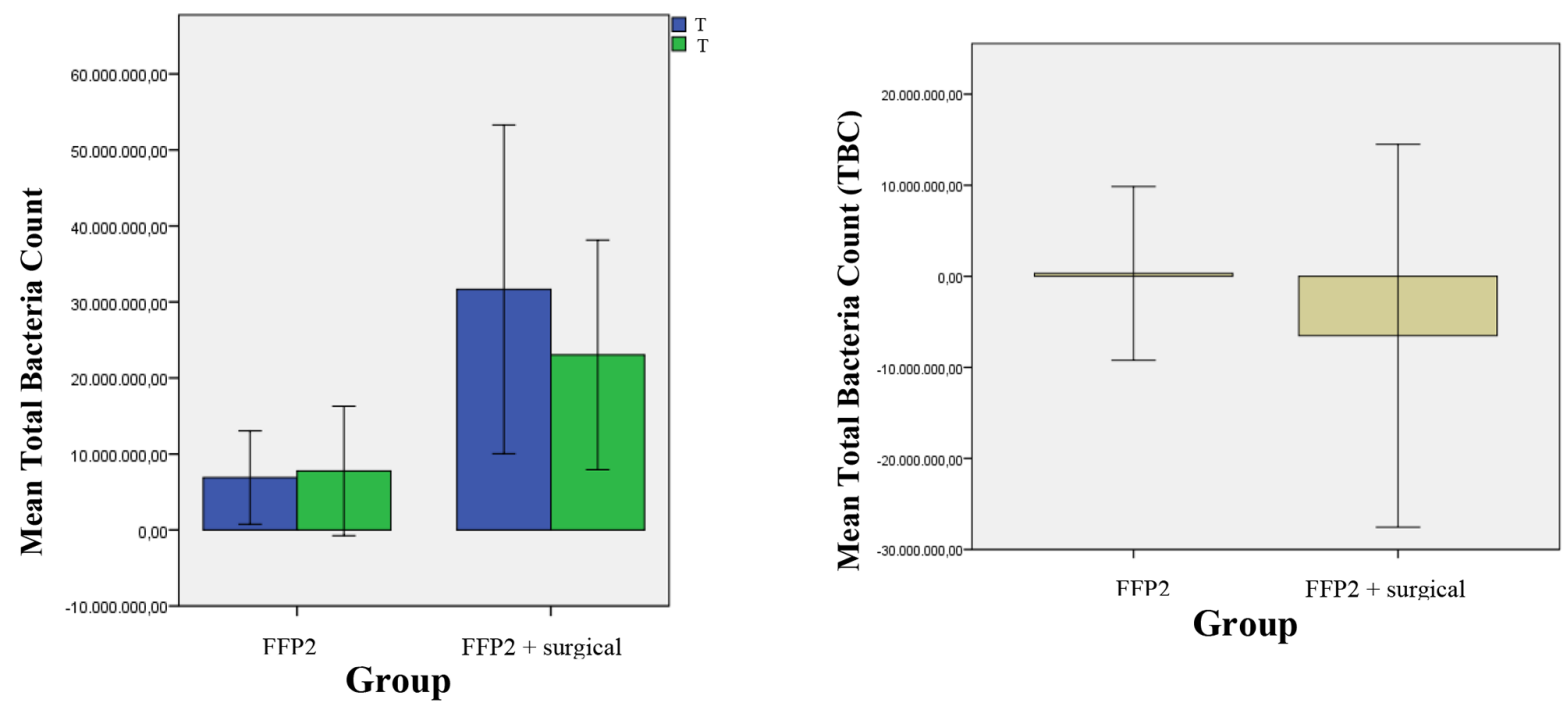
Mean of difference of T_1_-T_0_ and relative standard deviation of the TBC in 2 groups

Aerobic TBC did not change in group A after three hours of wearing an FFP2 mask (Fig. 9). The simultaneous use of FFP2 and surgical masks in group B, caused a significant decrease in TBC between T_0_ and T_1_. The difference between the T_0_/T_1_ average indicates that combining the two masks affects the TBC, unlike the FFP2 mask alone.

At baseline, no *Candida* spp were found on Group A, contrary to group B.

**Fig. 10:**
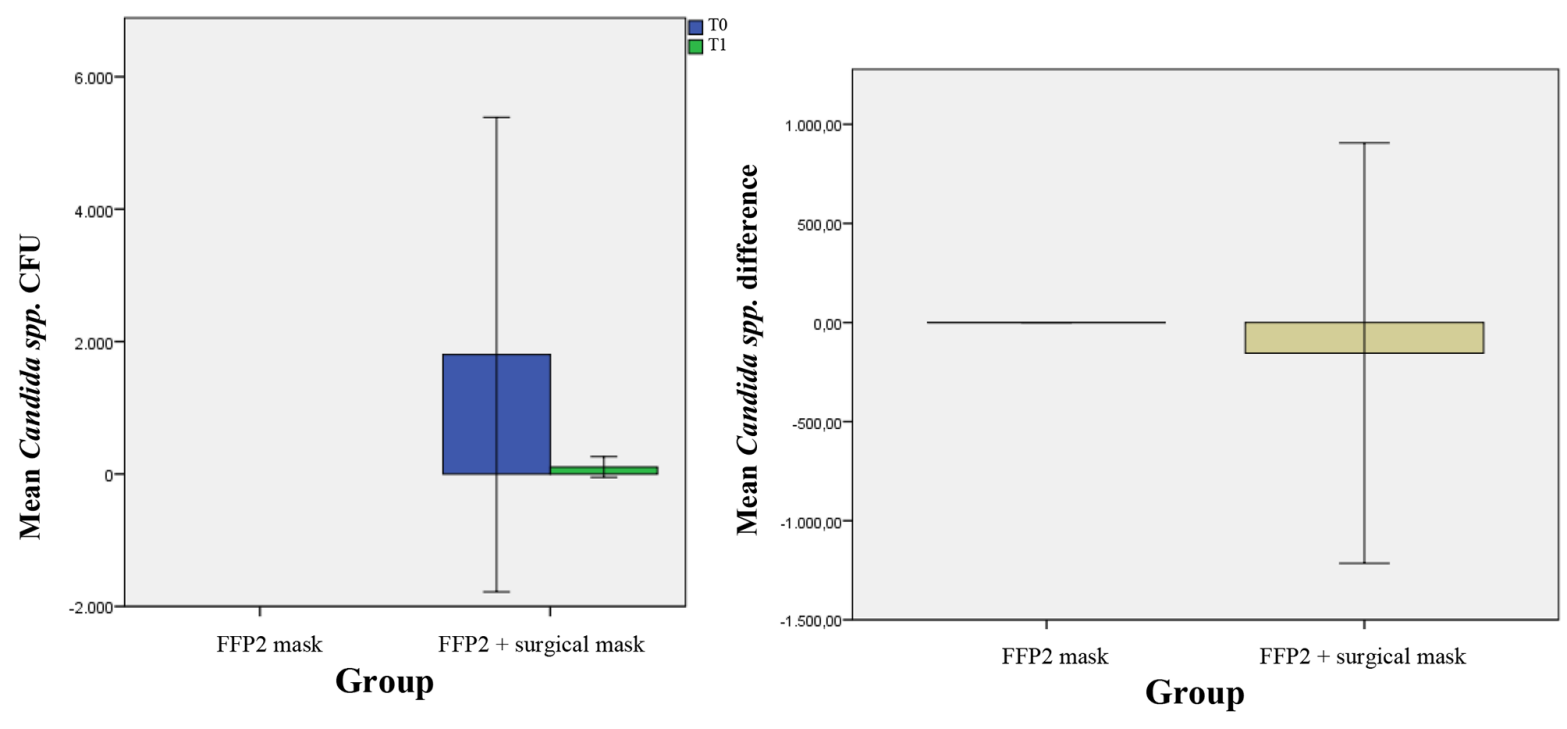
Mean of difference of T_1_-T_0_ and relative standard deviation of the *Candida spp.* CFU in 2 groups

Results of the presence of *Candida* spp, later identified as *Candida albicans* showed the absence of positive volunteers in group A, in contrast with 6 in group B (Fig. 10). The combined use of FFP2 and surgical masks results in a significant reduction in *Candida albicans* CFUs.

## Discussion

The COVID-19 pandemic irreversibly marked the last years, from 2019 until today, leading to drastic solutions, such as quarantine and radical changes in the population’s lifestyle [7, 24, 25]. As a form of prevention, we started to use masks, surgical or FFP2; currently, despite not being mandatory, the use continues in common places [26, 27]. The figure of today’s health professionals is allied to the constant use of the FFP2 mask in the clinic: the Doctor and Dentist must, also for social reasons, use the FFP2 mask during the work shift, guaranteeing patients new and higher standards of hygiene and protection [26]. During the exercise of the profession, it is rare for health professionals to remove masks, especially during long-term interventions. The additional coverage of the FFP2 mask with the surgical mask has the function of greater protection and hygiene, as it allows only the latter to be replaced between one patient and another and psychologically gives a greater sense of security in the transmission of the virus [26, 28].

Based on the achieved results, the null hypothesis tested had to be rejected because statistically significant differences were observed when the participants wearing masks with two different modalities. The results of this work highlight that the combined use of the two masks increases the individual problems even more, confirming the alternative hypothesis.

This preliminary study was born from the hypothesis that PPE, by creating a seal in the nose and the mouth, increase its concentration at the site by altering the exchange of carbon dioxide and its diffusion in the air, increased its concentration at the site. In the oral cavity, the increase in the % of CO_2_ is reflected in the resident microbiota, favoring the growth of anaerobic bacterial species to the detriment of aerobic ones and therefore, promoting pathogenic clusters of the periodontium [24]. In addition, CO_2_ reacting with saliva H_2_O produces carbonic acid (H_2_CO_3_), and lowers pH levels, facilitating enamel demineralization and favoring a possible carious process [15].

The oral microbiota contained within the confines of the masks holds the potential to precipitate dermatological complexities. A research undertaken by Park et al. 2020 [29] was centered on the meticulous examination of dermatological transformations engendered by mask usage, along with the dynamics of these changes over the temporal progression of the COVID-19 pandemic. The findings unveiled that subsequent to intervals of 1 and 6 h following mask donning, discernible alterations were witnessed encompassing skin temperature, the onset of erythema, levels of cutaneous hydration, and the secretion of sebum. As delineated within the investigation conducted by Huo et al. 2021 [9], instances where air accumulates betwixt the mask and the visage are likely to engender a notable augmentation in carbon dioxide (CO_2_) concentration in comparison to the naturally exhaled air. In this context, such an occurrence, particularly during inhalation, may be correlated with an elevated proclivity toward episodes of headache, as discerned from the outcomes of their study. Likewise, the study conducted by Lee et al. 2023 [30] delineates how the accumulation of CO_2_ can culminate in escalated pulmonary ventilation and heightened respiratory activity. This, in turn, can potentially lead to variations in skin temperature and humidity parameters.

34 volunteers accepted to participate in this study, in which they were randomly divided into 2 groups: group A with 17 participants and group B with 17. The inclusion criteria were respected since the participants were between 24 and 38 years old and did not present any previous or current history of relevant medical and/or dental illnesses. The volunteers were dental surgeons and dentistry students trained in oral hygiene care. During the study, two of the participants was excluded from group B, due to discomfort and difficulty in prolonged mask use, then the results are based on 32 participating volunteers.

DMFT index values confirmed mean lows of 2.37 ± 1.21, similar to the Italian average for the adult population. According to WHO estimates in “Oral Health Surveys: Basic Methods”, DMFT values below 5 in adults are considered a healthy oral cavity. However, this index reflects the oral health of the participants, as only 15.6% of the total had enamel caries in a single tooth, while fillings increased the DMF index, on average 2.06 ± 0.84 per volunteer.

Continuous use of the FFP2 mask for 3 h is a source of discomfort, leading to headaches, increased temperature at the site, shortness of breath, and other problems [10, 12, 24, 31]. In confirmation, two volunteers from group B were excluded from this research due to improper use and continuous removal of the PPE.

Without a proper fit, the degree of airway protection provided by eyewear is reduced. The Fit-test is recommended by national and international bodies to ensure the correct fit of filtering facepieces for individual healthcare professionals [2, 7].

The limit of a facemask study is to obtain the tightness recommended by the manufacturers. Thus, in this study, a practical demonstration was carried out in which the participants respected the INAIL recommendations on using masks and the fit verification methods.

The results obtained by group A show a slight increase in mean VOCs and a slight decrease in rehydration times. The volume and pH of basal saliva remain almost unchanged, as do the volume and pH of stimulated saliva. Aerobic TBC remains constant on average, and all volunteers tested negative for *Candida* spp.

The results obtained by group B suggest that covering the FFP2 mask with a surgical mask significantly blocks the passage of VOCs more than FFP2 alone, increasing their concentration in the oral cavity and generating greater variations in the oral ecosystem. On average, there was a significant increase in VOCs and a non-statistically significant decrease in rehydration times. The real time breath analysis represents a consistent methods for the early diagnosis of the upper and lower tract respiratory, upper digestive system and oral disease [32]. The dental literature correlated a consistently higher VOCs level in accordance to the bacterial activity and metabolites release in the oral cavity in accordance to local flogosis foci, tooth decay, periodontal disease and peri-implantitis [33]. Conversely, one of the weak point is determined by the low specificity of the assessment in order to determine the VOCs alteration origin. In addition, several independent variables such as hormonal fluctuations, systemic diseases (such as diabetic ketoacidosis) and comorbidities need to be considered as potential confounding factors for these scopes [34]. The amount and pH of basal and stimulated saliva increased compared to group A, but without statistical significance. Regarding aerobic TBC, the decrease between the means at T_0_ and T_1_ is statistically significant. Finally, although the participants’ distribution occurred randomly, 6 of the 15 participants of group B were positive for *Candida albicans*, whose T_1_ CFU levels suffered a statistically significant decrease.

Therefore, comparing the two groups, it is clear that the simultaneous use of a surgical mask and FFP2 for three hours leads to greater variations in the oral ecosystem than the use of FFP2 alone. In particular, in group B there is a significant increase in volatile organic substances and basal saliva volume and a decrease in aerobic bacteria, probably in favor of anaerobic bacteria. These data fully agree with each other and confirm the thesis, according to which the improper use of masks, prolonged over time, determines negative variations in the ecological factors of the oral cavity [35].

As explicated within the research endeavors conducted by Arroyo et al. 2018 [36], it becomes evident that oral microbial entities possess a notable capacity to secure their adhesion upon diverse substrates inherent to the oral milieu. This inherent attribute endows them with the capability to intermingle with the resident microbiota, consequently fostering their establishment and perpetuation. Of particular note is the fact that distinct bacterial species within the oral milieu may manifest varied propensities for adherence and proliferation upon the inner surface of masks.

Subsequent to the meticulous inquiry undertaken by Lee et al. 2023 [37], a comprehensive scrutiny of the bacterial profile located upon the inner facet of masks came to light. This discerning investigation unveiled a robust association between the microbial constituents dwelling within saliva and those encompassing the oral cavity, thereby rendering this connection manifestly significant. The amassed data further corroborated that the quantity and diversity of oral microorganisms residing within the mask fabric denoted a distinct and bespoke configuration, emblematic of the oral microbiome’s singular nature. Upon scrutinizing the interior interface of the masks, the discernible presence of both Gram-negative and Gram-positive bacterial entities was incontestably discerned. Notably, the hierarchy of abundance for these microbial inhabitants adhered to the following sequence: *P. gingivalis* > *F. nucleatum* > *P. nigrescens* > *E. corrodens* > *T. forsythia* > *T. denticola* and *L. casei* > *P. micra* > *E. nodatum*.

Building upon the foundations of the study by Lee et al. 2023 [30], a salient connection was unveiled between the escalated presence of *P. gingivalis* confined within the mask’s confines and a concomitant elevation in the concentrations of volatile sulfide compounds. This class of compounds is recognized for its pivotal role in halitosis. Such a link was conspicuously established in tandem with the presence of other salivary bacteria, specifically *T. denticola*, *T. forsythia*, *P. intermedia*, and *P. nigrescens*. Moreover, an additional study, as reported by Lin et al. [38] in the same year, spotlighted the unequivocal nexus between *P. gingivalis* and *F. nucleatum* with the advent of xerostomia following the administration of radioactive iodine therapy.

FFP2 masks comprise several layers and allow filtering between 94 and 99% of particles with a size between 0.01 and 150 μm in diameter [2, 28]. Thus, avoid the passage of bacteria, dust, and especially the SARS-CoV-2 virus that is transported by droplets with a diameter > 5 μm. On the other hand, carbon dioxide has a diameter of 116 nm, smaller than the texture of FFP2, thus allowing its passage. Theoretically, therefore, masks can filter the air we inhale and have the ability to expel carbon dioxide [2, 3, 28, 39].

Scarano et al. [10], demonstrated that the FFP2 mask produces a greater increase in facial temperature than the surgical mask, accompanied by greater discomfort. Inevitably, the situation worsens if a surgical mask is used over it as demonstrated in the present work. In dental surgeons who wear a FFP2 covered by a surgical mask for a long time, there is a reduction of blood % of O_2_ without clinical relevance, while an increase in heart rate, sensation of shortness of breath of breath, dizziness and headache [9, 11].

The authors demonstrated that a FFP2 mask, covered by a surgical mask, causes discomfort in breathing, decreased mental and physical performance, fatigue, especially during long operations, and can cause high levels of CO_2_ [8, 10, 11], as confirmed in our work.

In another study, a significant increase in local CO_2_ concentrations was demonstrated with the use of an FFP2 mask. Therefore, the use of valved masks that induce lower levels is recommended [12].

Studies designed to correlate O_2_ and CO_2_ with the physical symptoms of health professionals are scarce, however, further studies with pulse oximetry are needed to determine the level of hypercapnia in relation to headaches and impaired work ability [28].

Furthermore, drawing insights from the extant literature, it emerges that the detriments stemming from prolonged mask usage transcend the ambit of prior elucidation, encompassing not only exposed factors but also considerations of the mask’s constituent materials and potential contact with residual substances. As underscored by the research conducted by Torres-Agullo et al. 2021 [40], face masks are predominantly composed of polypropylene, a material which harbors the latent risk of entrained microplastics, thereby positing a potential pathway for microplastic inhalation. Equally noteworthy is an observation from Han et al. 2021 [41], wherein the detachment of fibers characterized by minute dimensions, along with fragments and particles staunchly adhering to the inner strata of face masks, was discerned.

Expanding the purview, interconnected contaminants such as polycyclic aromatic hydrocarbons (PAHs), VOCs, and phthalates, hitherto identified within face masks, manifest the potential for desorption, which in turn could engender subsequent health ramifications. Such implications extend to the realm of complications like reproductive toxicity and the provocation of genetic mutations [30, 42, 43]. Expounding upon this discourse, the research by Jin et al. 2021 [43] revealed the ubiquity of diethyl phthalete (DEP) across all scrutinized face masks. Additionally, phthalate esters, specifically DEP and di-n-butyl phthalate (DBP), were established as noteworthy constituents, comprising an aggregate of roughly 85% of compounds encompassed within this category. The ascertained masks, culled from diverse global locales, exhibited variances in the concentrations of volatile organic compounds of intermediate to high volatility. Notably, naphthalene, a compound designated as a potential human carcinogen by the United States Environmental Protection Agency (USEPA) in 1998, was detected across the entire cohort of 60 face masks appraised, constituting approximately 80% of the collective mass of Polycyclic Aromatic Hydrocarbons (PAHs).

Equally germane, research by Wang et al. 2022 [44] pivoted upon the identification of phthalates within a subset of 12 surgical masks. The outcomes garnered therein underscored a conspicuous diversity in phthalate levels, spanning a gamut that extended from 55 ± 35 to 1700 ± 140 ng/mask. Of noteworthy import, the assessments proffered evidence indicating that din-butyl phthalate (DnBP) and di-2-ethylhexyl phthalate (DEHP) made substantial contributions, encapsulating a proportion varying from 42 to 100% of the total mass reservoir of phthalic esters.

In summary, it is evident that the prolonged use of PPE involves systemic changes in the user. The innovation of this preliminary study is that no articles in the literature link the prolonged use of masks to the oral ecosystem. The results obtained should be considered as an introduction to the realization of new studies, analyzing qualitative parameters of the oral microbiota. Having demonstrated an increase in VOCs and basal saliva volume and a decrease in aerobic TBC in participants with a double mask confirms an alteration in the homeostasis of the oral cavity with possible consequent dysbiosis.

The results presented are preliminary data. Further trials are underway to analyze certain anaerobic, putative cariogenic, and pathogenic bacterial species of the periodontium to confirm that using PPE, especially inappropriately for long periods, is one of the risk factors for infectious diseases of the oral cavity.

This investigation yields significant contributions to the prevailing body of literature by delving into the nexus between the utilization of diverse protective mask types and the state of the oral ecosystem. Through direct elucidation of the ramifications of prolonged mask employment, this study addresses an existing lacuna by accentuating the plausible repercussions on oral well-being. Moreover, the comprehensive evaluation of multiple mask types coupled with consideration of parameters encompassing humidity, microbiota, and volatile organic compounds engenders a comprehensive assessment of intricate interplays. By affording equal attention to advantages and prospective hazards, the study furnishes an impartial panorama that has the potential to guide public health policy, shape judicious mask usage recommendations, and foster a greater comprehension of the repercussions of mask utilization within the contemporary milieu.

Therefore, we recommend to dentists and all healthcare professionals that preventive measures are necessary, not only to stop the spreading of SARS-CoV-2, but should be used with full knowledge of the facts and at the same time special attention and care should be taken provided the proper hygiene and health of the oral cavity in order to avoid harmful consequences.

Nonetheless, it is imperative to acknowledge certain intrinsic constraints inherent to this study that may exert an influence on the interpretation of findings. The employed sample might not comprehensively encompass the spectrum of mask varieties and individual attributes across divergent demographics, thereby potentially circumscribing the generalizability of the deductions. Additionally, the limited duration of the study may not capture the long-term effects of continued mask use. Uncontrolled variables, inclusive of individual proclivities in oral hygiene practices and the application of oral care commodities, may engender ambiguity within the outcomes, thereby rendering a direct ascription of the observed effects exclusively to mask utilization a challenge. The interplay with other plausible precipitating factors possibly remains inadequately accounted for, as does the sway exerted by individual conduct vis-à-vis mask adherence. Consequently, due cognizance of these limitations becomes requisite while delineating the implications of the outcomes and whilst extrapolating their implications to wider precincts, which imposes the need for future studies.

## Conclusion

According to the methodology applied and the results obtained, it is concluded that the prolonged use of the FFP2 mask covered by a surgical mask can generate oral and systemic changes in the user.

## Data Availability

The complete data and materials described in the research article are freely available from the corresponding author on reasonable request.
